# Trait impulsivity and risk of cardiovascular disease over 8 years: results from the NutriNet-Santé cohort

**DOI:** 10.1007/s10654-026-01383-5

**Published:** 2026-03-18

**Authors:** Carlos Gómez-Martínez, Pauline Paolassini-Guesnier, Léopold K. Fezeu, Bernard Srour, Serge Hercberg, Mathilde Touvier, Nancy Babio, Jordi Salas-Salvadó, Sandrine Péneau

**Affiliations:** 1 Universitat Rovira i Virgili, Departament de Bioquímica i Biotecnologia, Grup d’Alimentació, Nutrició, Desenvolupament i Salut Mental (ANUT-DSM), Unitat de Nutrició Humana, Reus, Spain; 2 Centro de Investigación Biomédica en Red de Fisiopatología de la Obesidad y Nutrición (CIBEROBN), Instituto de Salud Carlos III (ISCIII), Madrid, Spain; 3 Institut d’Investigació Sanitària Pere Virgili (IISPV), Grup d’Alimentació, Nutrició, Desenvolupament i Salut Mental (ANUT-DSM), Reus, Spain; 4 Université Sorbonne Paris Nord and Université Paris Cité, INSERM, INRAE, CNAM, Centre for Research in Epidemiology and StatisticS (CRESS), Nutritional Epidemiology Research Team (EREN), F-93017 Bobigny, France; 5 Division of Psychiatry, Haukeland University Hospital, Bergen, Norway; 6 Department of Biomedicine, University of Bergen, 5009 Bergen, Norway

**Keywords:** Cardiovascular disease, Cerebrovascular disease, Coronary heart disease, Impulsivity

## Abstract

Cardiovascular disease (CVD) remains the leading global cause of death, despite being partially preventable. Emerging evidence suggests psychological traits, such as trait impulsivity, may influence disease onset. However, associations between impulsivity and CVD remain understudied. This study investigates associations between trait impulsivity and CVD incidence. We conducted a prospective analysis within the French NutriNet-Santé cohort between May 2014 (time of impulsivity assessment) and February 2023, including adults aged ≥ 18 years without prevalent CVD. Data were collected via a web-based platform. Trait impulsivity was assessed using the Barratt Impulsiveness Scale 11 and categorized as low, moderate (reference), or high. Incident CVD events, including coronary heart disease and cerebrovascular disease, were identified through follow-up assessments and confirmed by NutriNet-Santé experts using medical records. Multivariable Cox proportional hazards models estimated hazard ratios and 95% confidence intervals (HR [95%CI]). Potential interactions, such as prevalence of type 2 diabetes (T2D), were assessed. Among 48,135 participants (78.1% women; mean age: 50.5 ± 14.5 years), 1,184 developed CVD over a median follow-up period of 7.84 years (IQR: 4.04–8.50). High impulsivity was associated with increased CVD risk (HR = 1.27 [1.01, 1.59], *P* = 0.039), compared to moderate impulsivity. Among participants with T2D (n = 1,301), low impulsivity was associated with reduced CVD risk (HR = 0.42 [0.20, 0.88], *P* = 0.022); no such association was observed in those without T2D (*P* for interaction = 0.014). Higher trait impulsivity was associated with greater CVD risk, while lower impulsivity exhibited protection in individuals with T2D. Trait impulsivity may represent a relevant psychological risk factor for CVD and could inform prevention strategies.

**Supplementary Information:**

The online version contains supplementary material available at 10.1007/s10654-026-01383-5.

## Introduction

Cardiovascular disease (CVD) claimed 17.9 million lives globally in 2019, accounting for 32% of all deaths [1]. Recognized risk factors include genetics, tobacco use, poor diet, dyslipidemia, hypertension, and diabetes [2]. Personality traits, which define patterns of thoughts, feelings, and behaviors, have also been proposed as contributing factors in CVD onset [3]. Among these, impulsivity has received considerable attention.

Impulsivity emerges from a dynamic interaction between genetic and environmental influences, constituting a multidimensional construct encompassing both personality traits and behaviors [4, 5]. The personality trait of impulsivity is characterized by reduced attentional control, a tendency to act without forethought, difficulty inhibiting emotionally driven behavioral responses, challenges in considering future consequences, and a propensity for sensation seeking [6]. Although personality traits are generally established early in life and remain stable across contexts, impulsivity is comparatively more susceptible to change over the lifespan [5].

Trait impulsivity has been associated with non-communicable diseases, including obesity and type 2 diabetes (T2D) [7, 8]. Only one cross-sectional study has examined facets of impulsivity and a cardiometabolic risk composite, finding that impulsivity was associated with higher risk via distinct lifestyle pathways [9]. Additional studies have found trait impulsivity to be associated with higher smoking, food addiction, and body mass index (BMI), and poorer adherence to healthy diets [7, 10–12], factors related to CVD risk [2]. These findings suggest that high trait impulsivity may increase CVD risk, but no evidence exists on this relationship.

This study aimed to assess associations between trait impulsivity and 8-year incident CVD risk in the NutriNet-Santé cohort, as well as potential moderating effects by diet quality, BMI, and T2D prevalence. We hypothesized that higher impulsivity would increase the risk of incident CVD events.

## Methods

### Study design and population

A prospective study design was performed within the NutriNet-Santé cohort, a web-based observational study that aims to assess the associations between diet, its determinants, and health [13]. Recruitment started in May 2009 and is currently ongoing with open enrolment. Volunteers are recruited via multimedia campaigns targeting the general French population. Inclusion criteria is age ≥ 18 years, fluent in French, and internet access. Participants are followed via personal accounts on the study website (https://etude-nutrinet-sante.fr/), where they provided detailed information by completing several questionnaires.

At enrolment, participants complete self-administered web-based questionnaires on socioeconomic conditions, health status, and lifestyle characteristics including diet, physical activity, and anthropometrics. Thereafter, participants complete these questionnaires annually or biannually for weight, diet, and health, with additional optional questionnaires sent monthly. Further protocol details are available at: https://info.etude-nutrinet-sante.fr/siteinfo/, where comprehensive information on the rationale, design and methodology of the study is available. The study is registered at https://www.clinicaltrials.gov/ (NCT03335644). Patients or members of the public were not involved in the design, conduct, or reporting of this research.

All participants provided electronic informed consent before enrollment. The study was approved by the Institutional Review Board of the French Institute for Health and Medical Research (IRB INSERM no: 0000388FWA00005831) and the Commission Nationale de l’Informatique et des Libertés (CNIL no: 908450 and 909216). The study complies with the Declaration of Helsinki standards.

### Impulsivity

Trait impulsivity was assessed using the 11th version of the Barratt Impulsiveness Scale (BIS-11) [6], adapted from the validated French version of the BIS-10 [14]. The administration of this questionnaire was carried out between May and November 2014. The BIS-11 is a 30-item self-report questionnaire scored on a 4-point Likert scale ranging from “rarely/never” (1 point) to “almost always/always” (4 points). The BIS-11 total score ranges from 30 to 120 points and is obtained by summing its items, with higher scores indicating greater trait impulsivity. The α Cronbach coefficient for the total score was 0.77, indicating an acceptable internal consistency. Impulsivity categories were defined according to BIS-11 guidelines: low (< 52), moderate (52–71), and high (> 71) trait impulsivity [6].

### Ascertainment of cardiovascular disease events

Incident CVD was ascertained by integrating data from medical records provided by participants or physicians and information from different official national registers. Participants reported major health events, including CVD, via the annual health questionnaire, a biannual special check-up questionnaire, or anytime through their NutriNet-Santé account. They were also asked to submit medical records, such as diagnoses, examinations, hospitalizations, medication use, radiological reports, electrocardiograms, and angioplasty procedures. Study physicians validated these records and, when needed, obtained additional information by contacting participants’ physicians or medical institutions providing treatment. If no updates were provided for over a year on the study website, the research team contacted participants, families, or physicians to verify health status. Furthermore, CVD events were linked to the Système National d’Information Inter-Régimes de l’Assurance Maladie (SNIIRAM) database, administered by the Caisse Nationale de l’Assurance Maladie of the French national insurance system (CNAM), and the French national mortality registry (CépiDC) to identify CVD-related deaths. This linkage minimizes bias from unreported CVD events.

The CVD events were classified using the International Classification of Diseases 10th Revision (ICD-10) to obtain overall cardiovascular disease, coronary heart disease, and cerebrovascular disease. Coronary heart disease included: myocardial infarction (I21), acute coronary syndrome (I20.0 and I21.4), angina pectoris (I20.1, I20.8, I20.9), and angioplasty (Z95.8). Cerebrovascular disease included: stroke (I64) and transient ischemic attack (G45.8 and G45.9). Overall cardiovascular disease included both coronary heart and cerebrovascular diseases events. Additional details are provided in Supplementary Table 1.

### Covariates

Potential confounders of the association between trait impulsivity and CVD were collected. Sociodemographics included: sex, age (years), and educational level (less than high school, < 2 years after high school, ≥ 2 years after high school). Lifestyle included: smoking intensity (packs/day), physical activity (low, moderate, high) using the International Physical Activity Questionnaire (IPAQ), energy intake without alcohol (kcal/day), alcohol intake (g/day) and diet quality (simplified Programme National Nutrition Santé—Guidelines Score 2 (sPNNS-GS2). Anthropometrics included: BMI (kg/m2). Personal history of disease included prevalence or medication for hypertension (no, yes), hypercholesterolemia (no, yes), hypertriglyceridemia (no, yes), and T2D (no, yes), and depressive symptomatology (no, yes) assessed by the self-reported Center for Epidemiologic Studies Depression Scale (CES-D). Finally, family history of disease included: family history of CVD (no, yes).

### Statistical analysis

Participants from the NutriNet-Santé cohort who completed the impulsivity questionnaire and had no prevalent CVD at the impulsivity assessment were included. Covariates with missing values were handled using Multiple Imputation by Chained Equations (MICE) (Supplementary Method 1).

Baseline population characteristics were compared between included and excluded participants using t-test or chi-square test (Supplementary Table 2). The participants’ baseline characteristics are presented as numbers and percentages for qualitative variables, and as mean ± standard deviation (SD) for quantitative variables. Comparisons across trait impulsivity categories (low, moderate, high) were assessed using the chi-square test for categorical variables and ANOVA for quantitative variables.

Cox regression models, with hazard ratios and 95% confidence interval (HR 95%CI), were used to study the associations between trait impulsivity categories (low, moderate as the reference, and high) and the risk of CVD (overall, coronary heart, and cerebrovascular) incidence during follow-up. Incidence rates per 1,000 person-years were also estimated. *P* for difference was obtained using the Wald test to assess the global association between impulsivity categories and CVD. Participants contributed person-time from their impulsivity assessment to the date of the CVD event, date of last follow-up, date of death, or 8th February 2023, whichever occurred first. The main model was adjusted at baseline for demographic, lifestyle, dietary, and cardiometabolic factors. The rationale for covariate selection is detailed in Supplementary Table 3.

Linearity assumptions were evaluated using restricted cubic spline functions between trait impulsivity and overall, coronary heart, and cerebrovascular diseases (Supplementary Fig. 1; Supplementary Fig. 2; Supplementary Fig. 3, respectively). Visual inspection of the spline curves suggested that the associations were primarily driven by individuals with extreme impulsivity scores. Accordingly, the analyses of this work focused on studying the associations between categories of trait impulsivity and CVD risk, but linear associations between a 1SD increase in trait impulsivity and CVD risk were also assessed as supplementary analyses (Supplementary Table 4).

Cumulative hazard functions between impulsivity categories and the incidence of CVD were estimated (Supplementary Fig. 4). Pearson correlation coefficients were examined to confirm the absence of collinearity between continuous covariates (Supplementary Table 5). Schoenfeld residuals were evaluated to validate proportional hazard risk assumptions (Supplementary Table 6). Covariates violating the non-proportional hazard assumption were corrected by adding a logarithmic time interaction term (physical activity, energy intake excluding alcohol, and diet quality for overall CVD; and physical activity for cerebrovascular disease). In addition, Schoenfeld residuals were re-evaluated by correcting for covariates that showed a non-proportional hazard risk (Supplementary Table 6).

Sensitivity analyses were conducted to assess the robustness of the results. The association between impulsivity and overall CVD risk was analyzed using stratified estimates for non-proportional hazard covariates, instead of using a logarithmic time interaction, as well as without correction for non-proportional hazard risk covariates. A total of seven additional models were assessed to investigate potential confounding effects. Associations between trait impulsivity and hard CVD events were also evaluated. Hard coronary heart disease included myocardial infarction, acute coronary syndrome, and angioplasty, and excluded angina pectoris. Hard cerebrovascular disease included stroke and excluded transient ischemic attack. An additional analysis excluded CVD events occurring within the first 2 years of follow-up to address potential reverse causality. Interactions by sex, age (< 60y, ≥ 60y), overweight (BMI: < 25, ≥ 25 kg/m2), diet quality (sPNNS-GS2, dichotomized at the median), and T2D prevalence (no, yes) were tested using the likelihood ratio test. The only statistically significant interaction was observed with T2D. Accordingly, analyses for overall CVD were stratified by T2D status, while associations with coronary heart disease and cerebrovascular disease are presented in Supplementary Table 7 and Supplementary Table 8, respectively, due to the limited number of incident cases which may limit interpretability. Finally, we conducted an inverse probability weighting analysis to account for potential selection bias arising from differences between included and excluded participants.

Analyses were conducted using Stata 14 and statistical significance was defined as *P* < 0.05.

## Results

### Description of the study population

**Fig. 1:**
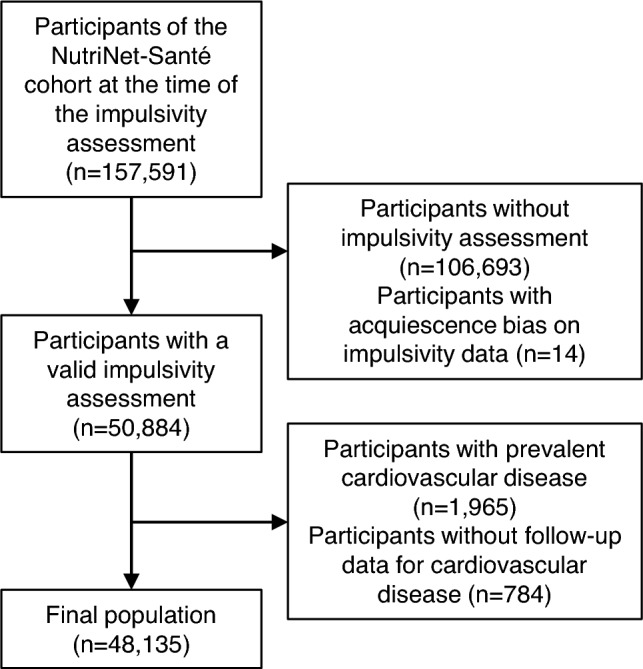
Flowchart of the studied population

Of the 157,591 participants enrolled in the NutriNet-Santé study at the time of the impulsivity assessment, 109,456 were excluded due to missing impulsivity data (non-mandatory questionnaire; n = 106,707), missing CVD data (n = 784), or prevalent CVD (n = 1,965) (Fig. 1). Among the 48,135 participants included, a total of 1,184 developed CVD during the median follow-up of nearly 8 years (median: 7.84; IQR: 4.04–8.50) (person-years: 292,769), of which 632 were classified as coronary heart disease and 554 as cerebrovascular disease (two participants developed both on the same day). The incidence rates (95%CI) per 1,000 person-years were 4.04 (3.82, 4.28) for overall CVD, 2.14 (1.98, 2.32) for coronary heart disease, and 1.88 (1.73, 2.04) for cerebrovascular disease. Frequencies and rates for each CVD subtype are provided in Supplementary Table 1.

Baseline characteristics of the population are presented in Table 1. The mean age of the population was 50.5 years (SD: 14.5), and approximately three-quarters were female. Individuals with higher impulsivity were more likely to be younger and female, have lower educational level, less physical activity, and poorer diet quality. They also exhibited higher smoking intensity, alcohol consumption and BMI, a higher prevalence or use of medication for hypertension, hypertriglyceridemia and T2D, and a higher prevalence of depressive symptomatology.

**Table 1:** Baseline characteristics of the study population, NutriNet-Santé cohort, France, 2014–2023 (n = 48,135)

Characteristics	All participants (n = 48,135)	Trait impulsivity			*P*-value*
		Low (n = 8,582)	Moderate (n = 36,577)	High (n = 2,976)	
Age (years)	50.47 ± 14.49^†^	50.93 ± 14.02	50.43 ± 14.53	49.68 ± 15.27	< 0.001
Sex (female)	37,580 (78.07)^‡^	6,233 (72.63)	28,881 (78.96)	2,466 (82.86)	< 0.001
Educational level (n = 47,985)					< 0.001
Less than high school	1,056 (2.20)	119 (1.39)	803 (2.20)	134 (4.51)	
< 2 years after high school	14,054 (29.29)	2,152 (25.14)	10,713 (29.39)	1,189 (40.03)	
≥ 2 years after high school	32,875 (68.51)	6,289 (73.47)	24,939 (68.41)	1,647 (55.45)	
Smoking intensity (packs/day) (n = 45,871)	0.30 ± 0.49	0.25 ± 0.45	0.30 ± 0.49	0.42 ± 0.56	< 0.001
Physical activity (IPAQ) (n = 48,037)					< 0.001
Low	11,084 (23.07)	1,865 (21.77)	8,453 (23.15)	766 (25.88)	
Moderate	20,143 (41.93)	3,541 (41.34)	15,420 (42.23)	1,182 (39.93)	
High	16,810 (34.99)	3,160 (36.89)	12,638 (34.61)	1,012 (34.19)	
Energy intake excluding alcohol (kcal/day) (n = 44,819)	1,787 ± 474	1,786 ± 473	1,786 ± 472	1,789 ± 502	0.44
Alcohol intake (g/day) (n = 44,819)	7.85 ± 12.00	7.05 ± 11.12	7.98 ± 12.07	8.63 ± 13.45	< 0.001
Diet quality (sPNNS-GS2; range: − 17 to 13.5) (n = 44,044)	1.27 ± 3.56	1.41 ± 3.55	1.26 ± 3.51	0.95 ± 3.78	< 0.001
BMI (kg/m^2^) (n = 47,962)	23.96 ± 4.50	23.71 ± 4.26	23.98 ± 4.49	24.55 ± 5.16	< 0.001
Hypertension prevalence or medication	6,412 (13.32)	1,117 (13.02)	4,840 (13.23)	455 (15.29)	0.004
Hypercholesterolemia prevalence or medication	8,601 (17.87)	1,498 (17.46)	6,549 (17.90)	554 (18.62)	0.34
Hypertriglyceridemia prevalence or medication	1,827 (3.80)	310 (3.61)	1,366 (3.73)	151 (5.07)	0.001
T2D prevalence or medication	1,301 (2.70)	206 (2.40)	982 (2.68)	113 (3.80)	< 0.001
Family history of CVD (n = 47,850)	17,529 (36.63)	3,090 (36.19)	13,355 (36.72)	1,084 (36.90)	0.63
Depressive symptomatology (n = 19,393)	2,358 (12.16)	263 (7.79)	1,799 (12.11)	296 (25.45)	< 0.001

*IPAQ* International Physical Activity Questionnaire, *sPNNS-GS2* simplified Programme National Nutrition Santé—Guidelines Score 2, *BMI* body mass index, *T2D* type 2 diabetes, *CVD* cardiovascular disease

Trait impulsivity categories were determined using the following cut-offs: low (< 52), moderate (≥ 52 and ≤ 71) and high (> 71), based on the Barratt Impulsiveness Scale 11 questionnaire

^*^ P-value showing comparisons between categories of trait impulsivity (low, moderate, high) based on chi-square for categorical variables and ANOVA for quantitative variables

^†^ mean ± SD (all such values)

^‡^ n (%) (all such values)

### Associations between trait impulsivity and CVD

**Fig. 2:**
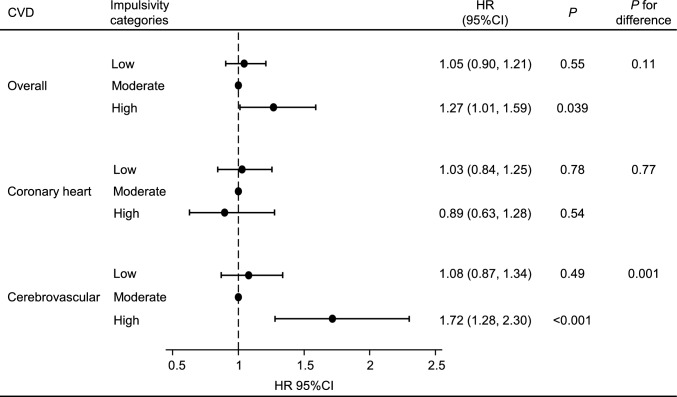
Associations between trait impulsivity and cardiovascular disease risk. NutriNet-Santé cohort, France, 2014–2023 (n = 48,135). *CVD* cardiovascular disease, *HR (95%CI)* hazard ratio and 95% confidence interval, *T2D* type 2 diabetes rate, incidence rate per 1,000 person-years, *T2D* type 2 diabetes. Incident cases and rates per 1,000 person-years for overall CVD (n = 1,184/48,135; rate = 4.04), coronary heart disease (n = 632/48,112; rate = 2.14), and cerebrovascular disease (n = 554/48,123; rate = 1.88). Cox regression analyses were performed using hazard ratios and 95%CI to assess associations between impulsivity categories (moderate as reference) and the risk of developing overall CVD, coronary heart disease, and cerebrovascular disease over a median follow-up of 8 years. The *P* for difference was obtained by using the Wald test to assess the global association between impulsivity categories and the respective CVD. Main model was adjusted for baseline age (time-scale), sex, educational level (less than high school degree, < 2 years after high school degree, ≥ 2 years after high school degree), smoking intensity (packs/day), physical activity (International Physical Activity Questionnaire: low, moderate, high), energy intake excluding alcohol (kcal/day), alcohol intake (g/day), diet quality (simplified Programme National Nutrition Santé—Guidelines Score 2), BMI (kg/m2), hypertension prevalence or medication (no, yes), hypercholesterolemia prevalence or medication (no, yes), hypertriglyceridemia prevalence or medication (no, yes), and T2D prevalence or medication (no, yes)

Compared with participants with moderate impulsivity, those with high impulsivity had an increased risk of developing overall CVD (HR [95%CI] = 1.27 [1.01,1.59]; *P* = 0.039), particularly cerebrovascular disease (HR = 1.72 [1.28, 2.30]; *P* < 0.001) (Fig. 2). No significant associations were found for coronary heart disease. Unadjusted estimates showed similar coefficients and unchanged statistical significance. High impulsivity was associated with increased risk of overall CVD (HR = 1.28 [1.02–1.60]; *P* = 0.032) and cerebrovascular disease (HR = 1.71 [1.28–2.29]; *P* < 0.001), but not with coronary heart disease.

### Associations between trait impulsivity and CVD by T2D prevalence

**Fig. 3:**
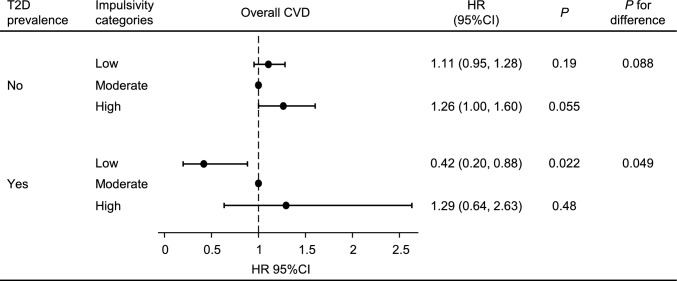
Associations between trait impulsivity and cardiovascular disease risk by type 2 diabetes prevalence. NutriNet-Santé cohort, France, 2014–2023 (n = 48,135). *T2D* type 2 diabetes, *CVD* cardiovascular disease, *HR (95%CI)* hazard ratio and 95% confidence interval. Participants without prevalent T2D: incident cases and rates per 1,000 person-years for overall CVD (n = 1,096/46,834; rate = 3.84). Participants with prevalent T2D: incident cases and rates per 1,000 person-years for overall CVD (n = 88/1,301; rate = 11.91). Cox regression analyses were performed using hazard ratios and 95%CI to assess associations between impulsivity categories (moderate as reference) and the risk of developing overall CVD over a median follow-up of 8 years in the NutriNet-Santé cohort. The *P* for difference was obtained by using the Wald test to assess the global association between impulsivity categories and overall CVD. Analyses were stratified by T2D prevalence due to a significant interaction (*P* = 0.014). Main model was adjusted for baseline age (time-scale), sex, educational level (less than high school degree, < 2 years after high school degree, ≥ 2 years after high school degree), smoking intensity (packs/day), physical activity (International Physical Activity Questionnaire: low, moderate, high), energy intake excluding alcohol (kcal/day), alcohol intake (g/day), diet quality (simplified Programme National Nutrition Santé—Guidelines Score 2), BMI (kg/m2), hypertension prevalence or medication (no, yes), hypercholesterolemia prevalence or medication (no, yes), and hypertriglyceridemia prevalence or medication (no, yes)

Analyses were stratified by T2D due to a significant interaction (*P* = 0.014) (Fig. 3). Among participants without T2D at baseline, those with high impulsivity had a borderline increased risk of developing overall CVD (*P* = 0.055), compared to those with moderate impulsivity. Among participants with prevalent T2D (n = 1,301), those with high impulsivity did not exhibit a significantly increased risk of CVD, whereas those with low impulsivity had a decreased risk of developing overall CVD (HR = 0.42 [0.20, 0.88]; *P* = 0.022). Additional analyses for coronary heart disease and cerebrovascular disease were conducted separately for individuals with and without T2D (Supplementary Table 7 & Supplementary Table 8, respectively).

### Sensitivity analyses

The correction using stratified estimates for covariates showing non-proportional hazard risk (Supplementary Table 9), as well as the non-correction of those covariates (Supplementary Table 10), confirmed the association between impulsivity and overall CVD risk. Associations between impulsivity and overall, coronary heart, and cerebrovascular diseases remained consistent across seven additional models controlling for various confounding factors (Supplementary Table 11). Furthermore, results from the inverse probability–weighted analysis were highly consistent with the main findings (Supplementary Table 12). However, the associations were attenuated and lost significance when restricted to hard CVD events (Supplementary Table 13) and after excluding events within the first 2 years of follow-up (Supplementary Table 14).

## Discussion

This study is the first to examine associations between trait impulsivity and CVD incidence. Our findings indicate that participants with higher impulsivity had an increased risk of developing CVD overall over 8 years of follow-up, particularly cerebrovascular events, compared with those with moderate impulsivity. Additionally, among individuals with prevalent T2D, those with lower impulsivity had a reduced risk of developing CVD overall.

Although no prior studies have specifically examined trait impulsivity and incident CVD, our findings align with a cross-sectional study reporting positive associations between impulsivity and a composite score of cardiometabolic risk [9]. Multiple mechanisms may contribute to the observed associations. First, trait impulsivity has been linked to greater propensity for food addiction and adherence to unhealthy dietary patterns [11, 12], the latter being a recognized contributing risk factor for CVD onset [2]. In addition, impulsivity has been positively associated with BMI and odds of hypertension, key contributors to cardiovascular risk [2, 7, 15]. Another potential mechanism is based on the mind-heart-body framework, which suggests that psychological factors, such as stress, interact with heart reactivity and hormonal release [16, 17]. Stress-related brain regions, including the limbic and prefrontal areas, have been associated with cardiovascular risk [18], and interestingly these neural regions also underlie brain areas involved in impulsivity [19]. Finally, the serotonin and dopamine networks, important regulators of impulsivity [19], have also been linked to cardiovascular function and may impact CVD development and prevention [20, 21].

In our longitudinal study, an association was observed between trait impulsivity and incident cerebrovascular disease, but not with coronary heart disease. To our knowledge, no prior studies have reported such a discrepancy. A pooled analysis of six United States cohorts including 58,105 participants found that conscientiousness and neuroticism, traits related to impulsivity [22], were inversely and positively associated with incident stroke, respectively [23]. However, their study did not assess coronary heart disease [23]. Contrary to our findings, a low internal locus of control, another construct related to impulsivity due to shared difficulties in self-regulation [19], was associated with higher myocardial infarction risk but showed no association with stroke [24].

The mechanisms underlying the association observed in our study between trait impulsivity and incident cerebrovascular disease, but not coronary heart disease, may reflect differential effects of impulsivity on cardiovascular risk factors and pathophysiology. Impulsivity has been associated with elevated blood pressure [15], which is a stronger risk factor for cerebrovascular disease than coronary heart disease [25]. This difference may be attributed to the inherent vulnerabilities of cerebral arteries, which have thinner walls and reduced elastic tissue compared to coronary arteries [26]. Moreover, impulsivity has been associated with increased use of alcohol, cigarettes, or amphetamines [10], behaviors having stronger associations with cerebrovascular disease than coronary heart disease [27–29].

T2D is a well-established risk factor for incident CVD [2]. In the present study, the relationship between higher impulsivity and increased CVD risk among individuals without T2D was similar to that observed in the full population, though it was not statistically significant. In the case of participants with T2D, higher impulsivity was also not significantly associated with CVD, but the limited statistical power in the T2D group may have masked a potentially elevated risk that warrants further investigation. However, low impulsivity was associated with a decreased risk of CVD, suggesting a protective effect. Some biological and behavioral mechanisms may help explain this pattern. Insulin resistance, hyperglycemia, chronic low-grade inflammation, oxidative stress, and endothelial dysfunction are key pathophysiological processes in both T2D and CVD [30], and several of these pathways have also been associated with higher impulsivity [31–34]. Consequently, lower impulsivity may reflect reduced exposure to these biological stressors, which could in turn lower CVD risk. Previous research within the same cohort showed a positive association between impulsivity and the incidence of T2D [8]. In individuals with T2D, facets of trait impulsivity have been linked to poorer self-care behaviors, including weight gain, reduced physical activity, poor diet quality, medication non-adherence, and poor diabetes control [31, 35–37]. These findings suggest that reducing trait impulsivity could be a promising strategy for decreasing incident CVD risk in individuals with T2D. However, the relatively small number of participants with T2D in this analysis warrants further research to confirm these findings.

Strengths of this study include the exploration of novel associations, a relatively large sample size and follow-up period, multiple assessments of CVD events, extensive sensitivity analyses, and the robustness of associations after adjusting for multiple confounders. However, several limitations exist. One limitation is the observational design, which precludes causal inference. Another limitation is that excluding incident cases within the first 2 years led to a loss of significance, which may be explained by reductions in CVD events by nearly half. This pattern may also indicate that early, undetected cardiovascular disease processes could have influenced baseline impulsivity scores. Although personality traits tend to be relatively stable in adulthood, trait impulsivity is more susceptible to change [5], and reverse causation cannot be fully ruled out. Sensitivity analyses restricted to hard CVD events also yielded non-significant results, probably due to reduced statistical power (e.g., cerebrovascular event incidence dropped from 1.1% to 0.3%). However, longer follow‑up with a greater number of hard CVD events will be essential to more reliably assess any potential association with impulsivity. In addition, trait impulsivity was self-reported, which may introduce bias despite the use of a widely accepted and validated questionnaire, and it was assessed only once, which limits the ability to capture potential changes over time. Selection bias should also be acknowledged, since the completion of the impulsivity questionnaire was optional. Nonetheless, analyses accounting for differential participation using inverse probability weighting suggested that the impact of this selection mechanism on our estimates was limited. The voluntary enrollment of NutriNet-Santé participants further constrains generalizability. A 2013 French study involving 3.5 million individuals reported CVD incidence rates exceeding those in our cohort, with notably higher rates in men than in women [38]. Given that our population is predominantly female, highly educated, and healthier than the general French population [39], it might be possible to observe a limited variability of impulsivity and incident events of CVD, by reducing scores and rates, respectively. However, the direction of bias cannot be determined with certainty. In addition, residual confounding factors, such as social determinants or medication adherence, may also play a role in the studied associations.

The clinical relevance of this study warrants emphasis. Although impulsivity traits can be adaptive in certain contexts [19], high impulsivity has been consistently related to a poor lifestyle and health status along with increased comorbidity of psychiatric conditions [9, 10, 12, 19, 36], contributing to a substantial public health burden. Notably, our study further found relationships between impulsivity and the leading cause of death worldwide, CVD [1], and that low impulsivity potentially has a protective effect in individuals with T2D. Within the mind-heart-body framework, efforts to reduce impulsivity have the potential to establish a positive feedback loop between enhanced psychological states and cardiovascular health [16].

In conclusion, participants with higher trait impulsivity had an increased risk of developing CVD over 8 years in a large French cohort, while those with T2D and lower impulsivity showed a decreased risk. Further prospective studies and clinical trials are needed to confirm these novel findings showing that trait impulsivity emerges as a promising psychological factor to consider in CVD prevention strategies.

## Supplementary Information

Below is the link to the electronic supplementary material.

Supplementary file1 (DOCX 398 KB)

## Data Availability

Researchers from public institutions may send a request for collaboration to Dr. Mathilde Touvier at: collaboration@etude-nutrinet-sante.fr, including information about their institution and a brief description of the proposed project. All requests will be reviewed by the NutriNet-Santé steering committee. If the collaboration is accepted, a data access agreement will be required, and appropriate authorizations from the competent administrative authorities may be needed. In accordance with current regulations, no personal data will be accessible. The analysis code can be requested from the authors.

## Abbreviations

BIS-11: 11Th version of the Barratt Impulsiveness Scale
BMI: Body mass index
CES-D: Center for epidemiologic studies depression scale
CNAM: Caisse Nationale de l’Assurance Maladie of the French national insurance
CVD: Cardiovascular disease
HR (95%CI): Hazard ratios and 95% confidence intervals
ICD-10: International Classification of Diseases 10th Revision
IPAQ: International physical activity questionnaire
IQR: Interquartile range
MICE: Multiple imputation by chained equations
SNIIRAM: Système national d’Information Inter-Régimes de l’Assurance Maladie database
SD: Standard deviation
sPNNS-GS2: Simplified programme national nutrition Santé-Guidelines Score 2
T2D: Type 2 diabetes
CépiDC: French national mortality registry
