# Supplementary material for: Trait impulsivity and risk of cardiovascular disease over 8 years: results from the NutriNet-Santé cohort
Source: Eur J Epidemiol. 2026 Mar 18;41(6):755–64. doi: 10.1007/s10654-026-01383-5 (PMC13423993; doi:10.1007/s10654-026-01383-5)
Supplement: Supplementary file 1 — Supplementary file1 (DOCX 398 KB) [file 10654_2026_1383_MOESM1_ESM.docx]

**Supplementary Method 1.** Multiple Imputation by Chained Equations.

Within the sample (n=48,135), missing values for covariates were handled using the Multiple Imputation by Chained Equations method using fully conditional specification (imputed datasets= 20; seed= 1234) for the following covariates: educational level (n=150 of missing data; 0.3% of missing data), smoking intensity pack-day (n=2,264; 4.7%), physical activity (n=98; 0.2%), energy intake excluding alcohol (n=3,316; 6.9%), alcohol intake (n=3,316; 6.9%), diet quality (n=4,091; 8.5%), BMI (n=173; 0.4%), family history of cardiovascular disease (CVD) (n=285; 0.6%), and depressive symptomatology (n=28,742; 59.7%).

**Supplementary Table 1.** Frequencies in incidence for each CVD subtype over 8 years with ICD-10 codes.

| CVD group | CVD subtype | ICD-10 code | Number of  incident events | Rate per 1,000 person-years |
| --- | --- | --- | --- | --- |
| Coronary heart disease | Myocardial infarction | I21 | 93 | 0.32 |
|  | Acute coronary syndrome | I20.0; I21.4 | 69 | 0.24 |
|  | Angina pectoris | I20.1; I20.8; I20.9 | 93 | 0.32 |
|  | Angioplasty | Z95.8 | 377 | 1.29 |
| Cerebrovascular disease | Stroke | I64 | 178 | 0.61 |
|  | Transient ischemic attack | G45.8; G45.9 | 376 | 1.28 |

n=48,135
Only the first event that occurred for each participant is shown.

**Supplementary Table 2.** Baseline characteristics of included and excluded population.

| Characteristics | Included population (n=48,135) | | Excluded population (n=109,456) | | *P-*value* |
| --- | --- | --- | --- | --- | --- |
|  | n | Values | n | Values |  |
| Age (years) | 48,135 | 50.47 ± 14.49^†^ | 109,384 | 44.28 ± 14.42 | <0.001 |
| Sex (female) | 48,135 | 37,580 (78.07)^‡^ | 109,384 | 84,913 (77.63) | 0.051 |
| Educational level | 47,985 |  | 103,734 |  | <0.001 |
| Less than high school degree |  | 1,056 (2.20) |  | 4,063 (3.92) |  |
| <2 years after high school degree |  | 14,054 (29.29) |  | 38,807 (37.41) |  |
| ≥2 years after high school degree |  | 32,875 (68.51) |  | 60,864 (58.67) |  |
| Smoking intensity (packs/day) | 45,871 | 0.30 ± 0.49 | 105,911 | 0.33 ± 0.49 | <0.001 |
| Physical activity (IPAQ) | 48,037 |  | 93,757 |  | <0.001 |
| Low |  | 11,084 (23.07) |  | 25,643 (27,35) |  |
| Moderate |  | 20,143 (41.93) |  | 38,005 (40.54) |  |
| High |  | 16,810 (34.99) |  | 30,109 (32.11) |  |
| Energy intake excluding alcohol (kcal/day) | 44,819 | 1,787.03 ± 473.75 | 69,379 | 1,799.98 ± 493.58 | 0.030 |
| Alcohol intake (g/day) | 44,819 | 7.85 ± 12.00 | 69,379 | 7.39 ± 12.53 | <0.001 |
| Diet quality (sPNNS-GS2; range: −17 to 13.5) | 44,044 | 1.27 ± 3.56 | 59,793 | 1.02 ± 3.55 | <0.001 |
| BMI (kg/m^2^) | 47,962 | 23.96 ± 4.50 | 104,729 | 24.38 ± 5.07 | <0.001 |
| Hypertension prevalence and/or medication | 48,135 | 6,412 (13.32) | 107,939 | 10,630 (9.85) | <0.001 |
| Hypercholesterolemia prevalence and/or medication | 48,135 | 8,601 (17.87) | 107,940 | 10,221 (9.47) | <0.001 |
| Hypertriglyceridemia prevalence and/or medication | 48,135 | 1,827 (3.80) | 107,393 | 2,395 (2.22) | <0.001 |
| T2D prevalence and/or medication | 48,135 | 1,301 (2.70) | 107,939 | 2,508 (2.32) | <0.001 |
| Family history of CVD | 47,850 | 17,529 (36.63) | 105,878 | 28,908 (27.30) | <0.001 |
| Depressive symptomatology | 19,393 | 2,358 (12.16) | 18,294 | 2,997 (16.38) | <0.001 |

Abbreviations: IPAQ, International Physical Activity Questionnaire; sPNNS-GS2, simplified Programme National Nutrition Santé - Guidelines Score 2; BMI, body mass index; T2D, type 2 diabetes; CVD, cardiovascular disease.

Trait impulsivity categories were determined using the following cut-offs: low (<52), moderate (≥52 and ≤71) and high (>71), based on the Barratt Impulsiveness Scale 11 questionnaire.

* *P*-value showing comparisons between included and excluded population based on chi-square for categorical variables and t-test for quantitative variables.

^†^ mean ± SD (all such values).

^‡^ n (%) (all such values).

**Supplementary Table 3.** Rationale for selected confounders.

| **Covariate** | **Coding of the variable** | **Relationship with impulsivity** | **Relationship with CVD** | **Method for collection / measurement** |
| --- | --- | --- | --- | --- |
| Age | Used as time scale in Cox models | Impulsivity decreases with aging ^1,2^ | Aging is associated with higher CVD risk ^3^ | Self-reported socio-demographic and lifestyle questionnaires |
| Sex | Categorical: male, female | There are different patterns of impulsivity between men and women ^4^ | Sex-related factors interact to produce differences in CVD outcomes ^5^ | Self-reported socio-demographic and lifestyle questionnaires |
| Educational level | Categorical: less than high school degree, <2 years after high school degree, ≥2 years after high school degree | Higher impulsivity is associated with lower educational level ^6^ | Higher educational level is associated with reduced CVD risk ^7^ | Self-reported socio-demographic and lifestyle questionnaires |
| Smoking intensity | Continuous (packs/day). A pack is defined as 20 cigarettes consumed in one day ^8^ | Higher impulsivity is associated with higher cigarette consumption ^9,10^ | Smoking packs/day is a better predictor than smoking status, smoking pack/years, and its combination to assess the risk of CVD outcomes ^8^ | Self-reported socio-demographic and lifestyle questionnaires |
| Physical activity | Categorical: low, moderate, high | Impulsivity is associated with patterns of physical activity ^11^ | Physical inactivity is associated with higher CVD risk ^3^ | Validated International Physical Activity Questionnaire ^12^ |
| Energy intake excluding alcohol intake | Continuous (kcal/day) | Higher impulsivity is associated with higher energy intake ^13^ | Higher energy intake is associated with higher CVD risk ^14^ | 24h-dietary records linked with the NutriNet-Santé food composition database |
| Alcohol intake | Continuous (g/day) | Higher impulsivity is associated with higher alcohol intake ^13^ | Light to moderate alcohol consumption is associated with reduced CVD risk, but heavy alcohol consumption is associated with higher CVD risk  ^15,16^ | 24h-dietary records linked with the NutriNet-Santé food composition database |
| Diet quality | Continuous: simplified Programme National Nutrition Santé - Guidelines Score 2 (range: −17 to 13.5) ^17^ | Higher impulsivity is associated with lower adherence to healthy dietary patterns and with higher adherence to unhealthy dietary patterns ^13,18^ | Poor diet quality is associated with higher CVD risk ^3,19^ | Validated 24h-dietary records linked with the NutriNet-Santé food composition database ^20^. sPNNS-GS2, simplified Programme National Nutrition Santé - Guidelines Score 2 ^17^ |
| BMI | Continuous (kg/m²) | Higher impulsivity is associated with higher BMI ^21^ | Obesity is associated with higher CVD risk ^22^ | Validated self-reported socio-demographic and lifestyle questionnaires ^23,24^ |
| Hypertension prevalence or medication | Categorical: yes, no | Higher impulsivity is associated with higher hypertension risk ^25^ | Hypertension is associated with higher CVD risk ^3^ | Self-reported socio-demographic and lifestyle questionnaires |
| Hypercholesterolemia prevalence or medication | Categorical: yes, no | Higher impulsivity is associated with lower HDL-cholesterol levels ^26^ | Hypercholesterolemia is associated with higher CVD risk ^3^ | Self-reported socio-demographic and lifestyle questionnaires |
| Hypertriglyceridemia prevalence or medication | Categorical: yes, no | Higher impulsivity is associated with higher triglyceride levels ^26^ | Hypertriglyceridemia is associated with higher CVD risk ^3^ | Self-reported socio-demographic and lifestyle questionnaires |
| T2D or medication (no, yes) | Categorical: yes, no | Impulsivity is higher in participants with T2D ^27^ | T2D is associated with higher CVD risk ^3^ | Self-reported socio-demographic and lifestyle questionnaires. Type 2 diabetes events were validated by the NutriNet-Santé physicians and further merged with French health institution data (SNIIRAM and CNAM) |
| **SENSITIVITY ANALYSES** | | | | |
| Family history of CVD | Categorical: yes, no | Impulsivity is associated with CVD risk ^28^. However, to our knowledge, no evidence exists regarding family history of CVD | Family history of CVD is associated with higher risk of CVD ^29^ | Self-reported socio-demographic and lifestyle questionnaires |
| Depressive symptomatology  (in sensitivity analyses due to the high number of missing data) | Categorical: yes, no | Higher impulsivity is associated with higher depressive symptomatology ^30^ | Depression is associated with higher CVD risk ^31^ | Self-reported Center for Epidemiologic Studies Depression Scale questionnaire ^32^ |

Abbreviations: CVD, cardiovascular disease; BMI, body mass index; T2D, type 2 diabetes; SNIIRAM, Système National d'Informations Inter-Régimes de l'Assurance Maladie; CNAM, Caisse nationale de l'Assurance Maladie.

**Supplementary Table 4.** Associations between 1SD increase in trait impulsivity and the risk of developing CVD over 8 years.

|  | CVD | HR (95% CI) | *P*-value |
| --- | --- | --- | --- |
| All participants | Overall | 1.03 (0.97, 1.09) | 0.34 |
| (n=48,135) | Coronary heart disease | 0.98 (0.90, 1.06) | 0.56 |
|  | Cerebrovascular disease | 1.09 (1.00, 1.18) | 0.052 |
| Absence of T2D | Overall | 1.01 (0.95, 1.07) | 0.81 |
| (n=46,834) | Coronary heart disease | 0.95 (0.87, 1.03) | 0.21 |
|  | Cerebrovascular disease | 1.23 (0.98, 1.56) | 0.079 |
| Prevalence of T2D | Overall | 1.29 (1.05, 1.57) | 0.015 |
| (n=1,301) | Coronary heart disease | 1.07 (0.98, 1.17) | 0.11 |
|  | Cerebrovascular disease | 1.41 (0.95, 2.09) | 0.091 |

Abbreviations: CVD, cardiovascular disease; HR (95%CI), hazard ratio and 95% confidence interval; T2D, type 2 diabetes.

Coronary heart disease includes: myocardial infarction, acute coronary syndrome, angioplasty, and angina pectoris. Cerebrovascular disease includes: stroke and transient ischemic attack. Overall CVD includes both coronary heart and cerebrovascular diseases.

Cox regression analyses were performed using hazard ratios and 95% CI to assess associations between 1SD increase in impulsivity and the risk of developing CVD, coronary heart disease, and cerebrovascular disease over a median follow-up of 8 years in the NutriNet-Santé cohort. Due to a significant T2D prevalence interaction (*P*=0.014), results were further stratified by T2D.

Main model was adjusted for baseline age (time-scale), sex, educational level (less than high school degree, <2 years after high school degree, ≥2 years after high school degree), smoking intensity (packs/day), physical activity (International Physical Activity Questionnaire: low, moderate, high), energy intake excluding alcohol (kcal/day), alcohol intake (g/day), diet quality (simplified Programme National Nutrition Santé - Guidelines Score 2), BMI (kg/m2), hypertension prevalence or medication (no, yes), hypercholesterolemia prevalence or medication (no, yes), hypertriglyceridemia prevalence or medication (no, yes), and T2D prevalence or medication (no, yes). Non-proportional hazard risk covariates were corrected by including a logarithmic time interaction term for physical activity, energy intake excluding alcohol, and diet quality in the analysis of overall CVD, and for physical activity in the analysis of cerebrovascular disease.

**Supplementary Table 5.** Pearson correlations between continuous variables included in the main model.

| Covariables | Age | Smoking intensity | Energy intake without alcohol | Alcohol intake | Diet quality | BMI |
| --- | --- | --- | --- | --- | --- | --- |
| Age | - |  |  |  |  |  |
| Smoking intensity | 0.22^§^* | - |  |  |  |  |
| Energy intake without alcohol | -0.030* | 0.0043* | - |  |  |  |
| Alcohol intake | 0.14* | 0.19* | 0.15* | - |  |  |
| Diet quality | 0.040* | -0.090* | -0.38* | -0.42* | - |  |
| BMI | 0.21* | 0.16* | 0.063* | 0.080* | -0.14* | - |

Abbreviations: BMI, body mass index.

^§^ Pearson correlation coefficients (all such values).

* *P*< 0.001

**Supplementary Table 6.** Assessment of proportional hazard risk assumptions between trait impulsivity and CVD incidence, with and without correction for non-proportional hazard risks covariates.

|  | Overall CVD | | Coronary heart | Cerebrovascular | |
| --- | --- | --- | --- | --- | --- |
|  | *P*-value | | *P*-value | *P*-value | |
| Characteristic | Without correction | With correction | Without correction | Without correction | With correction |
| Impulsivity |  |  |  |  |  |
| Low | 0.79 | 0.71 | 0.99 | 0.64 | 0.62 |
| Moderate | Ref. | Ref. | Ref. | Ref. | Ref. |
| High | 0.12 | 0.12 | 0.64 | 0.17 | 0.15 |
| Sex (female) | 0.13 | 0.087 | 0.052 | 0.97 | 0.97 |
| Educational level |  |  |  |  |  |
| Less than high school degree | 0.65 | 0.67 | 0.87 | 0.67 | 0.66 |
| <2 years after high school degree | Ref. | Ref. | Ref. | Ref. | Ref. |
| ≥2 years after high school degree | 0.96 | 0.78 | 0.57 | 0.51 | 0.50 |
| Smoking intensity (packs/day) | 0.24 | 0.28 | 0.064 | 0.93 | 0.91 |
| Physical activity |  |  |  |  |  |
| Low | Ref. | - | Ref. | Ref. | - |
| Moderate | 0.049 | - | 0.47 | 0.0021 | - |
| High | 0.71 | - | 0.15 | 0.11 | - |
| Energy intake without alcohol (kcal/day) | 0.039 | - | 0.072 | 0.32 | 0.26 |
| Alcohol intake (g/day) | 0.63 | 0.73 | 0.90 | 0.41 | 0.43 |
| Diet quality (sPNNS-GS2) | 0.018 | - | 0.064 | 0.14 | 0.14 |
| BMI (kg/m^2^) | 0.54 | 0.72 | 0.33 | 0.98 | 0.88 |
| Hypertension prevalence or medication | 0.29 | 0.19 | 0.92 | 0.11 | 0.13 |
| Hypercholesterolemia prevalence or medication | 0.35 | 0.33 | 0.68 | 0.33 | 0.35 |
| Hypertriglyceridemia prevalence or medication | 0.86 | 0.88 | 0.87 | 0.90 | 0.90 |
| T2D prevalence or medication | 0.17 | 0.17 | 0.20 | 0.45 | 0.43 |
| Global test | 0.055 | 0.40 | 0.36 | 0.14 | 0.52 |

Abbreviations: CVD, cardiovascular disease; sPNNS-GS2, simplified Programme National Nutrition Santé - Guidelines Score 2; BMI, body mass index; T2D, type 2 diabetes.

The assessment of proportional hazard risk was tested using the Schoenfeld residuals test in the main model.

The correction of non-proportional hazard risk was performed using stratified estimates for significant covariates showing a non-proportional hazard risk assumption at baseline (physical activity, energy intake excluding alcohol, and diet quality for overall CVD; physical activity for cerebrovascular disease).

**Supplementary Table 7.** Associations between trait impulsivity and risk of developing CVD over 8 years in participants without prevalence of type 2 diabetes.

|  | HR (95% CI) | *P*-value |  | Ref. | |  | HR (95% CI) | *P*-value |  | *P* for difference |
| --- | --- | --- | --- | --- | --- | --- | --- | --- | --- | --- |
| Coronary heart disease | Low impulsivity  (n=118/8,271) (rate=2.25) | |  | Moderate impulsivity (n=422/35,219) (rate=1.94) | |  | High impulsivity  (n=28/2,816) (rate=1.71) | |  |  |
|  | 1.11 (0.91, 1.37) | 0.30 |  | - | - |  | 0.87 (0.59, 1.28) | 0.48 |  | 0.41 |
| Cerebrovascular disease | Low impulsivity  (n=104/8,257) (rate=1.98) | |  | Moderate impulsivity  (n=375/35,172) (rate=1.72) | |  | High impulsivity  (n=47/2,835) (rate=2.89) | |  |  |
|  | 1.11 (0.89, 1.38) | 0.35 |  | - | - |  | 1.71 (1.26, 2.32) | 0.001 |  | 0.002 |

Abbreviations: CVD, cardiovascular disease; HR (95%CI), hazard ratio and 95% confidence interval; Ref, reference category; rate, incidence rate per 1,000 person-years; T2D, type 2 diabetes.

Coronary heart disease includes: myocardial infarction, acute coronary syndrome, angioplasty, and angina pectoris. Cerebrovascular disease includes: stroke and transient ischemic attack. Overall CVD includes both coronary heart and cerebrovascular diseases. The total number of participants, and the incident cases and incidence rates of CVDs, are described under each impulsivity category within the table.

Analyses were stratified by T2D prevalence due to a significant interaction (*P*=0.014). This table shows the results for participants without T2D at baseline. Cox regression analyses were performed using hazard ratios and 95% CI to assess associations between impulsivity categories (moderate as reference) and the risk of developing overall CVD (see Figure 2), coronary heart disease, and cerebrovascular disease over a median follow-up of 8 years in the NutriNet-Santé cohort. The *P* for difference was obtained by using the Wald test to assess the global association between impulsivity categories and the respective CVD.

Main model was adjusted for baseline age (time-scale), sex, educational level (less than high school degree, <2 years after high school degree, ≥2 years after high school degree), smoking intensity (packs/day), physical activity (International Physical Activity Questionnaire: low, moderate, high), energy intake excluding alcohol (kcal/day), alcohol intake (g/day), diet quality (simplified Programme National Nutrition Santé - Guidelines Score 2), BMI (kg/m2), hypertension prevalence or medication (no, yes), hypercholesterolemia prevalence or medication (no, yes), hypertriglyceridemia prevalence or medication (no, yes), and T2D prevalence or medication (no, yes). Non-proportional hazard risk covariates were corrected by including a logarithmic time interaction term for physical activity in the analysis of cerebrovascular disease.

**Supplementary Table 8.** Associations between trait impulsivity and risk of developing CVD over 8 years in participants presenting prevalence of type 2 diabetes.

|  | HR (95% CI) | *P*-value |  | HR (95% CI) | *P*-value |  | HR (95% CI) | *P*-value |  | *P* for difference |
| --- | --- | --- | --- | --- | --- | --- | --- | --- | --- | --- |
| Coronary heart disease | Low impulsivity  (n=6/204) (rate=4.66) | |  | Moderate impulsivity  (n=51/962) (rate=9.04) | |  | High impulsivity  (n=5/109) (rate=8.57) | |  |  |
|  | 0.41 (0.17, 1.97) | 0.043 |  | - | - |  | 0.96 (0.38, 2.45) | 0.93 |  | 0.13 |
| Cerebrovascular disease | Low impulsivity  (n=2/200) (rate=1.55) | |  | Moderate impulsivity  (n=20/931) (rate=3.46) | |  | High impulsivity  (n=4/108) (rate=6.88) | |  |  |
|  | 0.51 (0.12, 2.21) | 0.37 |  | - | - |  | 2.27 (0.73, 7.05) | 0.16 |  | 0.16 |

Abbreviations: CVD, cardiovascular disease; HR (95%CI), hazard ratio and 95% confidence interval; Ref, reference category; rate, incidence rate per 1,000 person-years; T2D, type 2 diabetes.

Coronary heart disease includes: myocardial infarction, acute coronary syndrome, angioplasty, and angina pectoris. Cerebrovascular disease includes: stroke and transient ischemic attack. Overall CVD includes both coronary heart and cerebrovascular diseases. The total number of participants, and the incident cases and incidence rates of CVDs, are described under each impulsivity category within the table.

Analyses were stratified by T2D prevalence due to a significant interaction (*P*=0.014). This table shows the results for participants having T2D at baseline. Cox regression analyses were performed using hazard ratios and 95% CI to assess associations between impulsivity categories (moderate as reference) and the risk of developing Overall CVD (see Figure 2), coronary heart disease, and cerebrovascular disease over a median follow-up of 8 years in the NutriNet-Santé cohort. The *P* for difference was obtained by using the Wald test to assess the global association between impulsivity categories and the respective CVD.

Main model was adjusted for baseline age (time-scale), sex, educational level (less than high school degree, <2 years after high school degree, ≥2 years after high school degree), smoking intensity (packs/day), physical activity (International Physical Activity Questionnaire: low, moderate, high), energy intake excluding alcohol (kcal/day), alcohol intake (g/day), diet quality (simplified Programme National Nutrition Santé - Guidelines Score 2), BMI (kg/m2), hypertension prevalence or medication (no, yes), hypercholesterolemia prevalence or medication (no, yes), hypertriglyceridemia prevalence or medication (no, yes), and T2D prevalence or medication (no, yes). Non-proportional hazard risk covariates were corrected by including a logarithmic time interaction term for physical activity in the analysis of cerebrovascular disease.

**Supplementary Table 9.** Associations between trait impulsivity and risk of developing CVD over 8 years using stratified estimates to account for non-proportional hazard risk covariates.

|  | HR (95% CI) | *P*-value |  | Ref. | |  | HR (95% CI) | *P*-value |  | *P* for difference |
| --- | --- | --- | --- | --- | --- | --- | --- | --- | --- | --- |
| Overall CVD | Low impulsivity  (n=231/8,582) (rate=4.29) | |  | Moderate impulsivity  (n=869/36,577) (rate=3.91) | |  | High impulsivity  (n=84/2,976) (rate=5.03) | |  |  |
|  | 1.05 (0.90, 1.21) | 0.55 |  | - | - |  | 1.27 (1.01, 1.60) | 0.037 |  | 0.10 |
| Cerebrovascular disease | Low impulsivity  (n=107/8,580) (rate=1.97) | |  | Moderate impulsivity  (n=396/36,567) (rate=1.77) | |  | High impulsivity  (n=51/2,976) (rate=3.03) | |  |  |
|  | 1.08 (0.87, 1.34) | 0.48 |  | - | - |  | 1.71 (1.27, 2.30) | <0.001 |  | 0.001 |

Abbreviations: CVD, cardiovascular disease; HR (95%CI), hazard ratio and 95% confidence interval; Ref, reference category; rate, incidence rate per 1,000 person-years; T2D, type 2 diabetes.

Cerebrovascular disease includes: stroke and transient ischemic attack. Overall CVD includes: myocardial infarction, acute coronary syndrome, angioplasty, angina pectoris, stroke, and transient ischemic attack. The total number of participants, and the incident cases and incidence rates of CVDs, are described under each impulsivity category within the table. Cox regression analyses were performed using hazard ratios and 95% CI to assess associations between impulsivity categories (moderate as reference) and the risk of developing overall CVD over a median follow-up of 8 years in the NutriNet-Santé cohort. *P* for difference was obtained by using the Wald test to assess the global association between impulsivity categories and the respective CVD.

Main model was adjusted for baseline age (time-scale), sex, educational level (less than high school degree, <2 years after high school degree, ≥2 years after high school degree), smoking intensity (packs/day), physical activity (International Physical Activity Questionnaire: low, moderate, high), energy intake excluding alcohol (kcal/day), alcohol intake (g/day), diet quality (simplified Programme National Nutrition Santé - Guidelines Score 2), BMI (kg/m2), hypertension prevalence or medication (no, yes), hypercholesterolemia prevalence or medication (no, yes), hypertriglyceridemia prevalence or medication (no, yes), and T2D prevalence or medication (no, yes). Non-proportional hazard risk covariates were corrected by using stratified estimates for physical activity, energy intake excluding alcohol, and diet quality in the analysis of overall CVD, and for physical activity in the analysis of cerebrovascular disease. Coronary heart disease was not included in this analysis as it did not present non-proportional hazard risk estimates.

**Supplementary Table 10.** Associations between trait impulsivity and risk of developing CVD over 8 years without correction for non-proportional hazard risk covariates.

|  | HR (95% CI) | *P*-value |  | Ref. | |  | HR (95% CI) | *P*-value |  | *P* for difference |
| --- | --- | --- | --- | --- | --- | --- | --- | --- | --- | --- |
| Overall CVD | Low impulsivity  (n=231/8,582) (rate=4.29) | |  | Moderate impulsivity  (n=869/36,577) (rate=3.91) | |  | High impulsivity  (n=84/2,976) (rate=5.03) | |  |  |
|  | 1.05 (0.90, 1.21) | 0.55 |  | - | - |  | 1.27 (1.01, 1.59) | 0.039 |  | 0.11 |
| Cerebrovascular disease | Low impulsivity  (n=107/8,580) (rate=1.97) | |  | Moderate impulsivity  (n=396/36,567) (rate=1.77) | |  | High impulsivity  (n=51/2,976) (rate=3.03) | |  |  |
|  | 1.08 (0.87, 1.34) | 0.49 |  | - | - |  | 1.72 (1.28, 2.30) | <0.001 |  | 0.001 |

Abbreviations: CVD, cardiovascular disease; HR (95%CI), hazard ratio and 95% confidence interval; Ref, reference category; rate, incidence rate per 1,000 person-years; T2D, type 2 diabetes.

Cerebrovascular disease includes: stroke and transient ischemic attack. Overall CVD includes: myocardial infarction, acute coronary syndrome, angioplasty, angina pectoris, stroke, and transient ischemic attack. The total number of participants, and the incident cases and incidence rates of CVDs, are described under each impulsivity category within the table.

Cox regression analyses were performed using hazard ratios and 95% CI to assess associations between impulsivity categories (moderate as reference) and the risk of developing overall CVD and cerebrovascular disease over a median follow-up of 8 years in the NutriNet-Santé cohort. *P* for difference was obtained by using the Wald test to assess the global association between impulsivity categories and the respective CVD.

Main model was adjusted for baseline age (time-scale), sex, educational level (less than high school degree, <2 years after high school degree, ≥2 years after high school degree), smoking intensity (packs/day), physical activity (International Physical Activity Questionnaire: low, moderate, high), energy intake excluding alcohol (kcal/day), alcohol intake (g/day), diet quality (simplified Programme National Nutrition Santé - Guidelines Score 2), BMI (kg/m2), hypertension prevalence or medication (no, yes), hypercholesterolemia prevalence or medication (no, yes), hypertriglyceridemia prevalence or medication (no, yes), and T2D prevalence or medication (no, yes). Coronary heart disease was not included in this analysis as it did not present non-proportional hazard risk estimates.

**Supplementary Table 11.** All models of associations between trait impulsivity and risk of developing CVD over 8 years.

|  |  | HR (95% CI) | *P*-value |  | Ref. | |  | HR (95% CI) | *P*-value |  | *P* for difference |
| --- | --- | --- | --- | --- | --- | --- | --- | --- | --- | --- | --- |
| Overall CVD |  | Low impulsivity  (n=231/8,582) (rate=4.29) | |  | Moderate impulsivity  (n=869/36,577) (rate=3.91) | |  | High impulsivity  (n=84/2,976) (rate=5.03) | |  |  |
|  | Model 1 | 1.02 (0.88, 1.18) | 0.80 |  | - | - |  | 1.34 (1.07, 1.67) | 0.011 |  | 0.040 |
|  | Model 2 | 1.03 (0.89, 1.19) | 0.70 |  | - | - |  | 1.29 (1.03, 1.62) | 0.025 |  | 0.080 |
|  | Model 3 | 1.03 (0.89, 1.19) | 0.73 |  | - | - |  | 1.29 (1.03, 1.61) | 0.029 |  | 0.090 |
|  | Model 4 | 1.03 (0.89, 1.19) | 0.73 |  | - | - |  | 1.28 (1.03, 1.61) | 0.029 |  | 0.090 |
|  | Model 5 | 1.03 (0.89, 1.19) | 0.67 |  | - | - |  | 1.27 (1.01, 1.59) | 0.038 |  | 0.11 |
|  | Model 6 | 1.05 (0.90, 1.21) | 0.55 |  | - | - |  | 1.27 (1.01, 1.59) | 0.038 |  | 0.12 |
|  | Model 7 | 1.05 (0.90, 1.21) | 0.53 |  | - | - |  | 1.27 (1.01, 1.59) | 0.042 |  | 0.12 |
| Coronary heart disease |  | Low impulsivity  (n=125/8,578) (rate=2.30) | |  | Moderate impulsivity  (n=474/36,558) (rate=2.12) | |  | High impulsivity  (n=33/2,976) (rate=1.95) | |  |  |
|  | Model 1 | 0.99 (0.81, 1.21) | 0.92 |  | - | - |  | 0.97 (0.68, 1.38) | 0.87 |  | 0.98 |
|  | Model 2 | 1.00 (0.82, 1.22) | 0.99 |  | - | - |  | 0.93 (0.65, 1.33) | 0.70 |  | 0.92 |
|  | Model 3 | 1.00 (0.82, 1.22) | 0.98 |  | - | - |  | 0.92 (0.65, 1.31) | 0.64 |  | 0.89 |
|  | Model 4 | 1.00 (0.82, 1.22) | 0.97 |  | - | - |  | 0.92 (0.64, 1.31) | 0.64 |  | 0.89 |
|  | Model 5 | 1.01 (0.83, 1.23) | 0.92 |  | - | - |  | 0.91 (0.64, 1.29) | 0.59 |  | 0.85 |
|  | Model 6 | 1.03 (0.84, 1.26) | 0.78 |  | - | - |  | 0.90 (0.63, 1.28) | 0.55 |  | 0.78 |
|  | Model 7 | 1.03 (0.85, 1.26) | 0.76 |  | - | - |  | 0.89 (0.63, 1.28) | 0.54 |  | 0.73 |
| Cerebrovascular disease |  | Low impulsivity  (n=107/8,580) (rate=1.97) | |  | Moderate impulsivity  (n=396/36,567) (rate=1.77) | |  | High impulsivity  (n=51/2,976) (rate=3.03) | |  |  |
|  | Model 1 | 1.07 (0.86, 1.32) | 0.56 |  | - | - |  | 1.75 (1.31, 2.35) | <0.001 |  | 0.001 |
|  | Model 2 | 1.07 (0.87, 1.33) | 0.51 |  | - | - |  | 1.72 (1.28, 2.30) | <0.001 |  | 0.001 |
|  | Model 3 | 1.06 (0.86, 1.32) | 0.58 |  | - | - |  | 1.72 (1.28, 2.31) | <0.001 |  | 0.001 |
|  | Model 4 | 1.06 (0.86, 1.32) | 0.58 |  | - | - |  | 1.72 (1.28, 2.31) | <0.001 |  | 0.001 |
|  | Model 5 | 1.07 (0.86, 1.32) | 0.55 |  | - | - |  | 1.71 (1.27, 2.29) | <0.001 |  | 0.001 |
|  | Model 6 | 1.08 (0.87, 1.34) | 0.52 |  | - | - |  | 1.72 (1.28, 2.30) | <0.001 |  | 0.002 |
|  | Model 7 | 1.08 (0.87, 1.34) | 0.48 |  | - | - |  | 1.72 (1.28, 2.31) | <0.001 |  | 0.002 |

Abbreviations: CVD, cardiovascular disease; HR (95%CI), hazard ratio and 95% confidence interval; Ref, reference category; rate, incidence rate per 1,000 person-years; T2D, type 2 diabetes.

Coronary heart disease includes: myocardial infarction, acute coronary syndrome, angioplasty, and angina pectoris. Cerebrovascular disease includes: stroke and transient ischemic attack. Overall CVD includes both coronary heart and cerebrovascular diseases. The total number of participants, and the incident cases and incidence rates of CVDs, are described under each impulsivity category within the table. Two participants had a coronary heart and cerebrovascular disease event on the same day, and these participants were included in both analyses.

Cox regression analyses were performed using hazard ratios and 95% CI to assess associations between impulsivity categories (moderate as reference) and the risk of developing overall CVD, coronary heart disease, and cerebrovascular disease over a median follow-up of 8 years in the NutriNet-Santé cohort. The *P* for difference was obtained by using the Wald test to assess the global association between impulsivity categories and the respective CVD. All confounding factors were adjusted at baseline in the following models.

Model 1: adjusted for sex and age (time-scale).

Model 2 (sociodemographics): Model 1 + educational level (less than high school degree, <2 years after high school degree, ≥2 years after high school degree).

Model 3 (lifestyle): Model 2 + smoking intensity (packs/day), physical activity (International Physical Activity Questionnaire: low, moderate, high), energy intake excluding alcohol (kcal/day), and alcohol intake (g/day).

Model 4 (diet quality): Model 3 + baseline diet quality (simplified Programme National Nutrition Santé - Guidelines Score 2).

Model 5 (anthropometrics): Model 4 + baseline body mass index (kg/m^2^).

Main model (personal history of disease): Model 5 + baseline hypertension prevalence or medication (no, yes), hypercholesterolemia prevalence or medication (no, yes), hypertriglyceridemia prevalence or medication (no, yes), and T2D prevalence or medication (no, yes). See Figure 2.

Model 6 (family history of disease): Main model + baseline family history of CVD (no, yes).

Model 7 (depressive symptomatology): Model 6 + baseline depressive symptomatology (Center for Epidemiologic Studies Depression Scale: no, yes).

Non-proportional hazard risk covariates were corrected by including a logarithmic time interaction term for physical activity, energy intake excluding alcohol, and diet quality in the analysis of overall CVD, and for physical activity in the analysis of cerebrovascular disease.

**Supplementary Table 12.** Associations between trait impulsivity and risk of developing CVD using inverse probability weighting.

|  | HR (95% CI) | *P*-value |  | Ref. | |  | HR (95% CI) | *P*-value |  | *P* for difference |
| --- | --- | --- | --- | --- | --- | --- | --- | --- | --- | --- |
| Overall CVD | Low impulsivity  (n=231/8,582) (rate=4.29) | |  | Moderate impulsivity  (n=869/36,577) (rate=3.91) | |  | High impulsivity  (n=84/2,976) (rate=5.03) | |  |  |
|  | 1.05 (0.90, 1.21) | 0.55 |  | - | - |  | 1.27 (1.01, 1.60) | 0.043 |  | 0.12 |
| Coronary heart disease | Low impulsivity  (n=125/8,578) (rate=2.30) | |  | Moderate impulsivity  (n=474/36,558) (rate=2.12) | |  | High impulsivity  (n=33/2,976) (rate=1.95) | |  |  |
|  | 1.02 (0.83, 1.24) | 0.87 |  | - | - |  | 0.87 (0.60, 1.25) | 0.44 |  | 0.72 |
| Cerebrovascular disease | Low impulsivity  (n=107/8,580) (rate=1.97) | |  | Moderate impulsivity  (n=396/36,567) (rate=1.77) | |  | High impulsivity  (n=51/2,976) (rate=3.03) | |  |  |
|  | 1.07 (0.86, 1.32) | 0.55 |  | - | - |  | 1.69 (1.26, 2.28) | 0.001 |  | 0.002 |

Abbreviations: CVD, cardiovascular disease; HR (95%CI), hazard ratio and 95% confidence interval; Ref, reference category; rate, incidence rate per 1,000 person-years; T2D, type 2 diabetes.

Coronary heart disease includes: myocardial infarction, acute coronary syndrome, angioplasty, and angina pectoris. Cerebrovascular disease includes: stroke and transient ischemic attack. Overall CVD includes both coronary heart and cerebrovascular diseases. The total number of participants, and the incident cases and incidence rates of CVDs, are described under each impulsivity category within the table. Two participants had a coronary heart and cerebrovascular disease event on the same day, and these participants were included in both analyses.

Inverse probability weighting estimates were calculated to address baseline differences between included and excluded participants, with weights modeled using all covariates included in the main analysis. Cox regression analyses were performed using hazard ratios and 95% CI to assess associations between impulsivity categories (moderate as reference) and the risk of developing overall CVD, coronary heart disease, and cerebrovascular disease over a median follow-up of 8 years in the NutriNet-Santé cohort. The *P* for difference was obtained by using the Wald test to assess the global association between impulsivity categories and the respective CVD.

Main model was adjusted for baseline age (time-scale), sex, educational level (less than high school degree, <2 years after high school degree, ≥2 years after high school degree), smoking intensity (packs/day), physical activity (International Physical Activity Questionnaire: low, moderate, high), energy intake excluding alcohol (kcal/day), alcohol intake (g/day), diet quality (simplified Programme National Nutrition Santé - Guidelines Score 2), BMI (kg/m2), hypertension prevalence or medication (no, yes), hypercholesterolemia prevalence or medication (no, yes), hypertriglyceridemia prevalence or medication (no, yes), and T2D prevalence or medication (no, yes).

Non-proportional hazard risk covariates were corrected by including a logarithmic time interaction term for physical activity, energy intake excluding alcohol, and diet quality in the analysis of overall CVD, and for physical activity in the analysis of cerebrovascular disease.

**Supplementary Table 13.** Associations between trait impulsivity and risk of developing hard CVD over 8 years.

|  | HR (95% CI) | *P*-value |  | Ref. | |  | HR (95% CI) | *P*-value |  | *P* for difference |
| --- | --- | --- | --- | --- | --- | --- | --- | --- | --- | --- |
| Hard overall CVD | Low impulsivity  (n=141/4,492) (rate=2.63) | |  | Moderate impulsivity  (n=561/36,269) (rate=2.53) | |  | High impulsivity  (n=39/2,931) (rate=2.35) | |  |  |
|  | 0.97 (0.81, 1.17) | 0.78 |  | - | - |  | 0.92 (0.66, 1.27) | 0.60 |  | 0.85 |
| Hard coronary heart disease | Low impulsivity  (n=113/8,491) (rate=2.10) | |  | Moderate impulsivity  (n=419/36,264) (rate=1.89) | |  | High impulsivity  (n=28/2,931) (rate=1.68) | |  |  |
|  | 1.03 (0.84, 1.28) | 0.75 |  | - | - |  | 0.87 (0.59, 1.28) | 0.47 |  | 0.71 |
| Hard cerebrovascular disease | Low impulsivity  (n=28/8,486) (rate=0.52) | |  | Moderate impulsivity  (n=142/36,250) (rate=0.64) | |  | High impulsivity  (n=11/2,931) (rate=0.66) | |  |  |
|  | 0.79 (0.52, 1.18) | 0.25 |  | - | - |  | 1.04 (0.56, 1.93) | 0.90 |  | 0.50 |

Abbreviations: CVD, cardiovascular disease; HR (95%CI), hazard ratio and 95% confidence interval; Ref, reference category; rate, incidence rate per 1,000 person-years; T2D, type 2 diabetes.

Hard coronary heart disease includes: myocardial infarction, acute coronary syndrome, and angioplasty (exclude angina pectoris). Hard cerebrovascular disease includes: stroke (exclude transient ischemic attack). Hard overall CVD includes both hard coronary heart and hard cerebrovascular diseases. The total number of participants, and the incident cases and incidence rates of hard CVDs, are described under each impulsivity category within the table.

Cox regression analyses were performed using hazard ratios and 95% CI to assess associations between impulsivity categories (moderate as reference) and the risk of developing hard overall CVD, hard coronary heart disease, and hard cerebrovascular disease over a median follow-up of 8 years in the NutriNet-Santé cohort. The *P* for difference was obtained by using the Wald test to assess the global association between impulsivity categories and the respective hard CVD.

Main model was adjusted for baseline age (time-scale), sex, educational level (less than high school degree, <2 years after high school degree, ≥2 years after high school degree), smoking intensity (packs/day), physical activity (International Physical Activity Questionnaire: low, moderate, high), energy intake excluding alcohol (kcal/day), alcohol intake (g/day), diet quality (simplified Programme National Nutrition Santé - Guidelines Score 2), BMI (kg/m2), hypertension prevalence or medication (no, yes), hypercholesterolemia prevalence or medication (no, yes), hypertriglyceridemia prevalence or medication (no, yes), and T2D prevalence or medication (no, yes). Non-proportional hazard risk covariates were corrected by including a logarithmic time interaction term for physical activity, energy intake excluding alcohol, and diet quality in the analysis of hard overall CVD, and for physical activity in the analysis of hard cerebrovascular disease.

**Supplementary Table 14.** Associations between trait impulsivity and risk of developing CVD over 8 years excluding CVD events in the first 2 years of follow-up.

|  | HR (95% CI) | *P*-value |  | Ref. | |  | HR (95% CI) | *P*-value |  | *P* for difference |
| --- | --- | --- | --- | --- | --- | --- | --- | --- | --- | --- |
| Overall CVD | Low impulsivity  (n=155/7,421) (rate=2.94) | |  | Moderate impulsivity  (n=571/31,125) (rate=2.63) | |  | High impulsivity  (n=48/2,407) (rate=2.95) | |  |  |
|  | 1.07 (0.90, 1.28) | 0.45 |  | - | - |  | 1.14 (0.85, 1.53) | 0.39 |  | 0.56 |
| Coronary heart disease | Low impulsivity  (n=89/7,355) (rate=1.69) | |  | Moderate impulsivity  (n=321/30,875) (rate=1.48) | |  | High impulsivity  (n=22/2,381) (rate=1.36) | |  |  |
|  | 1.09 (0.86, 1.38) | 0.49 |  | - | - |  | 0.92 (0.59, 1.42) | 0.70 |  | 0.72 |
| Cerebrovascular disease | Low impulsivity  (n=66/7,332) (rate=1.26) | |  | Moderate impulsivity  (n=250/30,804) (rate=1.16) | |  | High impulsivity  (n=26/2,385) (rate=1.61) | |  |  |
|  | 1.06 (0.81, 1.39) | 0.68 |  | - | - |  | 1.41 (0.94, 2.12) | 0.095 |  | 0.23 |

Abbreviations: CVD, cardiovascular disease; HR (95%CI), hazard ratio and 95% confidence interval; Ref, reference category; rate, incidence rate per 1,000 person-years; T2D, type 2 diabetes.

Coronary heart disease includes: myocardial infarction, acute coronary syndrome, angioplasty, and angina pectoris. Cerebrovascular disease includes: stroke and transient ischemic attack. Overall CVD includes both coronary heart and cerebrovascular diseases. The total number of participants, and the incident cases and incidence rates of CVDs, are described under each impulsivity category within the table.

Cox regression analyses were performed using hazard ratios and 95% CI to assess associations between impulsivity categories (moderate as reference) and the risk of developing overall CVD, coronary heart disease, and cerebrovascular disease over a median follow-up of 8.19 (interquartile range: 5.85-8.54) years in the NutriNet-Santé cohort. Participants with a CVD event in the first 2 years of follow-up were excluded. The *P* for difference was obtained by using the Wald test to assess the global association between impulsivity categories and the respective hard CVD.

Main model was adjusted for baseline age (time-scale), sex, educational level (less than high school degree, <2 years after high school degree, ≥2 years after high school degree), smoking intensity (packs/day), physical activity (International Physical Activity Questionnaire: low, moderate, high), energy intake excluding alcohol (kcal/day), alcohol intake (g/day), diet quality (simplified Programme National Nutrition Santé - Guidelines Score 2), BMI (kg/m2), hypertension prevalence or medication (no, yes), hypercholesterolemia prevalence or medication (no, yes), hypertriglyceridemia prevalence or medication (no, yes), and T2D prevalence or medication (no, yes). Non-proportional hazard risk covariates were corrected by including a logarithmic time interaction term for physical activity, energy intake excluding alcohol, and diet quality in the analysis of overall CVD, and for physical activity in the analysis of cerebrovascular disease.

**Supplementary Figure 1.** Restricted cubic splines between trait impulsivity and risk of developing overall CVD.

**
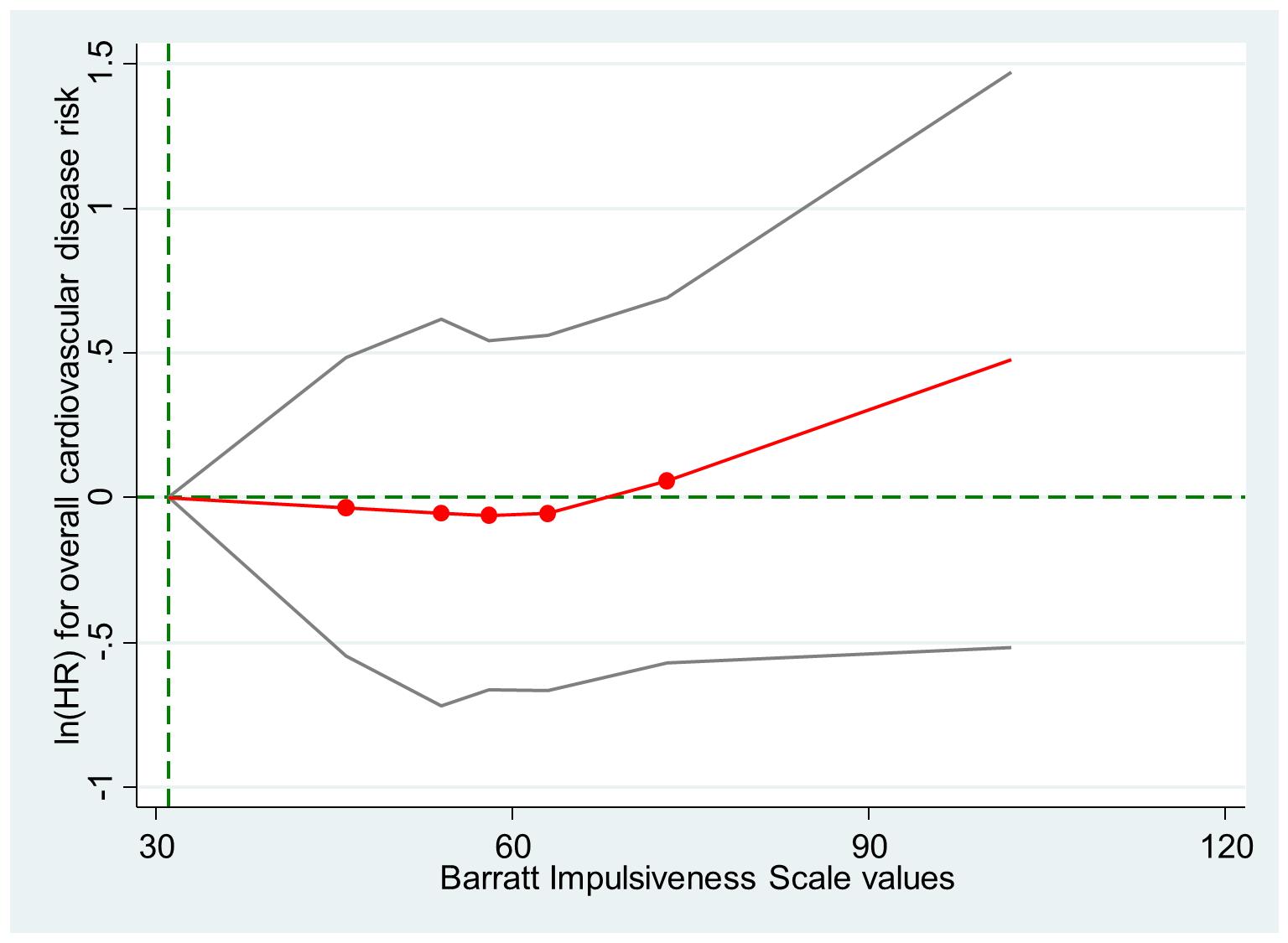
**

Cox regression analyses were performed using hazard ratios and 95% CI to assess associations between trait impulsivity values and the risk of developing overall CVD over a median follow-up of 8 years in the NutriNet-Santé cohort.

Spline plot modeling the association between total trait impulsivity and CVD risk was obtained using restricted cubic spline and piecewise cubic polynomials across 5 adjacent knots of trait impulsivity. Knots are represented with a red dot for total impulsivity: 46, 54, 58, 63, 73.

Main model was adjusted for baseline age (time-scale), sex, educational level (less than high school degree, <2 years after high school degree, ≥2 years after high school degree), smoking intensity (pack/day), physical activity (International Physical Activity Questionnaire: low, moderate, high), energy intake excluding alcohol (kcal/day), alcohol intake (g/day), diet quality (simplified Programme National Nutrition Santé - Guidelines Score 2), BMI (kg/m^2^), hypertension prevalence or medication (no, yes), hypercholesterolemia prevalence or medication (no, yes), hypertriglyceridemia prevalence or medication (no, yes), and T2D prevalence or medication (no, yes). *P*-value for potential non-linear association (*P*=0.65).

**Supplementary Figure 2.** Restricted cubic splines between trait impulsivity and risk of developing coronary heart disease.

**
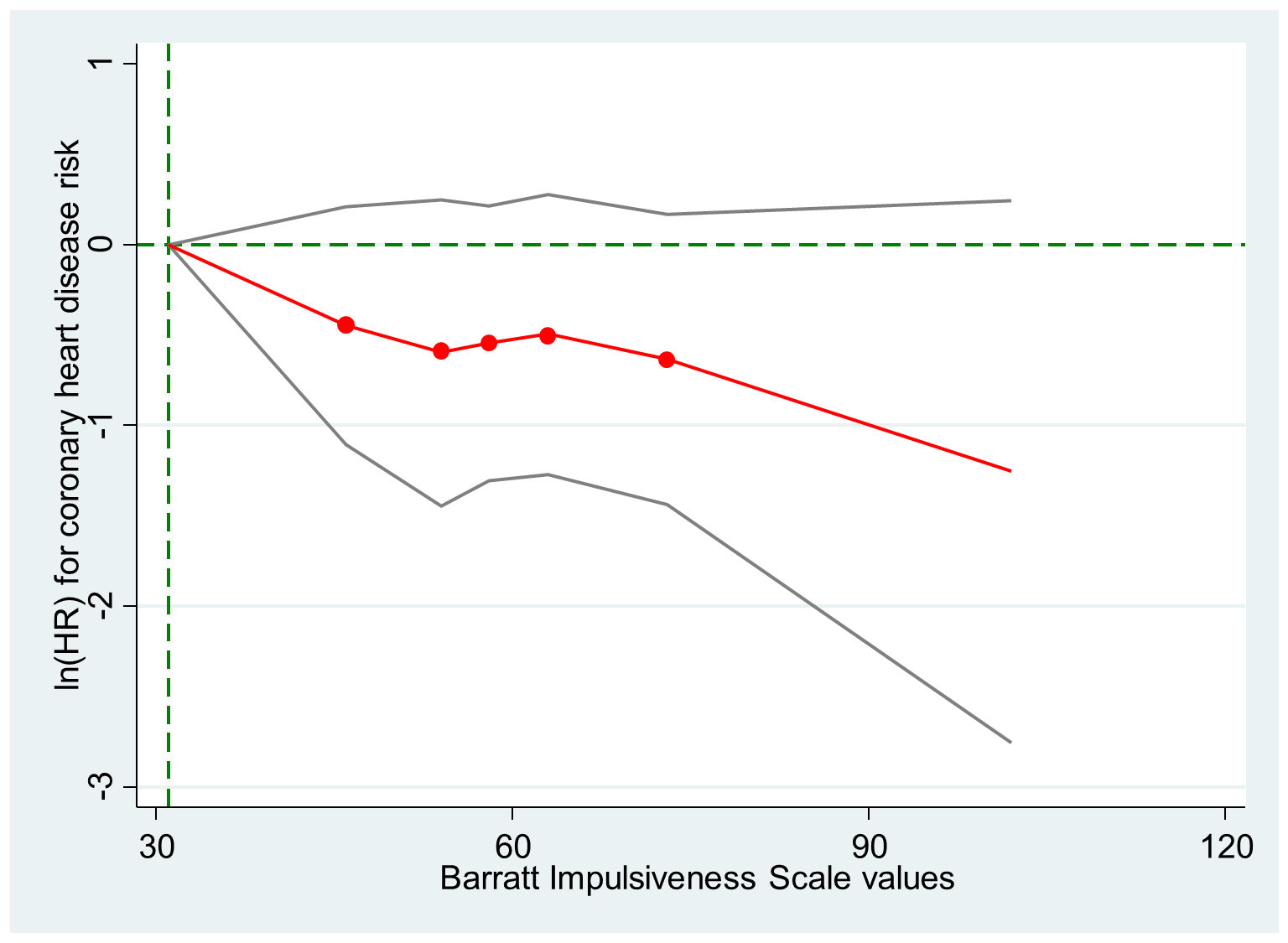
**

Cox regression analyses were performed using hazard ratios and 95% CI to assess associations between trait impulsivity values and the risk of developing coronary heart disease over a median follow-up of 8 years in the NutriNet-Santé cohort.

Spline plot modeling the association between total trait impulsivity and coronary heart disease risk was obtained using restricted cubic spline and piecewise cubic polynomials across 5 adjacent knots of trait impulsivity. Knots are represented with a red dot for total impulsivity: 46, 54, 58, 63, 73.

Main model was adjusted for baseline age (time-scale), sex, educational level (less than high school degree, <2 years after high school degree, ≥2 years after high school degree), smoking intensity (pack/day), physical activity (International Physical Activity Questionnaire: low, moderate, high), energy intake excluding alcohol (kcal/day), alcohol intake (g/day), diet quality (simplified Programme National Nutrition Santé - Guidelines Score 2), BMI (kg/m^2^), hypertension prevalence or medication (no, yes), hypercholesterolemia prevalence or medication (no, yes), hypertriglyceridemia prevalence or medication (no, yes), and T2D prevalence or medication (no, yes). *P*-value for potential non-linear association (*P*=0.49).

**Supplementary Figure 3.** Restricted cubic splines between trait impulsivity and risk of developing cerebrovascular disease.

**
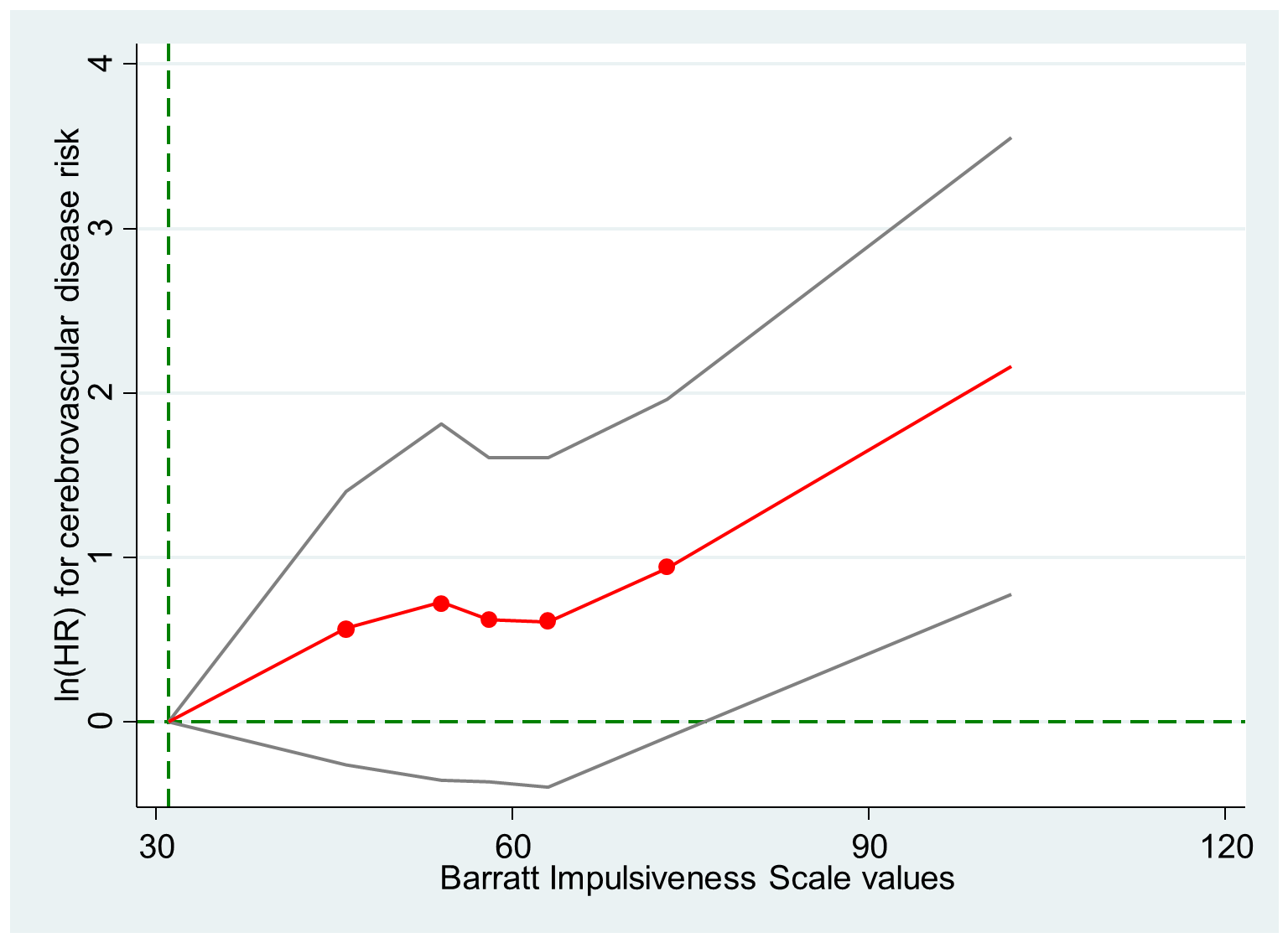
**

Cox regression analyses were performed using hazard ratios and 95% CI to assess associations between trait impulsivity values and the risk of developing cerebrovascular disease over a median follow-up of 8 years in the NutriNet-Santé cohort.

Spline plot modeling the association between total trait impulsivity and cerebrovascular disease risk was obtained using restricted cubic spline and piecewise cubic polynomials across 5 adjacent knots of trait impulsivity. Knots are represented with a red dot for total impulsivity: 46, 54, 58, 63, 73.

Main model was adjusted for baseline age (time-scale), sex, educational level (less than high school degree, <2 years after high school degree, ≥2 years after high school degree), smoking intensity (pack/day), physical activity (International Physical Activity Questionnaire: low, moderate, high), energy intake excluding alcohol (kcal/day), alcohol intake (g/day), diet quality (simplified Programme National Nutrition Santé - Guidelines Score 2), BMI (kg/m^2^), hypertension prevalence or medication (no, yes), hypercholesterolemia prevalence or medication (no, yes), hypertriglyceridemia prevalence or medication (no, yes), and T2D prevalence or medication (no, yes). *P*-value for potential non-linear association (*P*=0.030).

**Supplementary Figure 4.** Cumulative hazard ratio between categories of trait impulsivity and the risk of developing overall CVD.

**
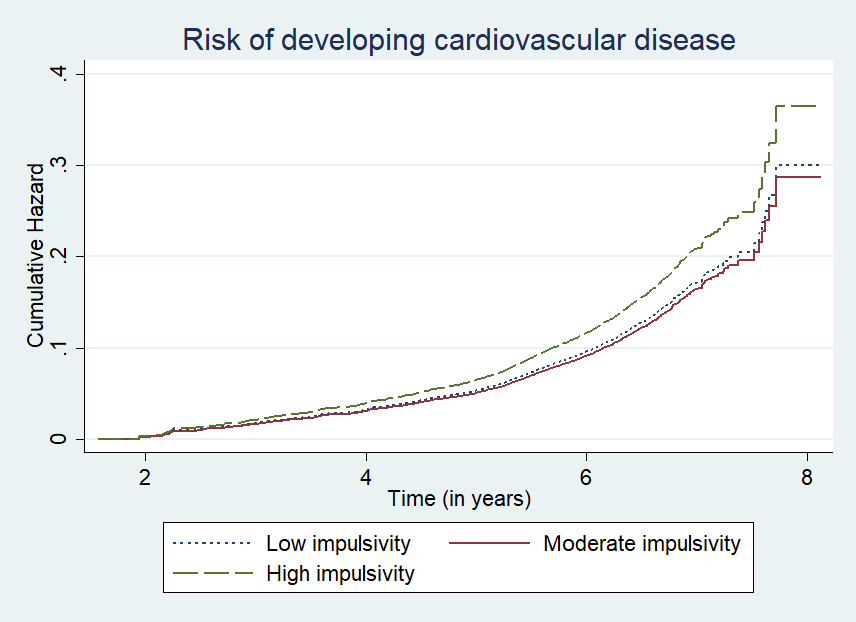
**

Cox regression analyses were performed using hazard ratios and 95% CI to assess associations across trait impulsivity categories and the risk of developing overall CVD over a median follow-up of 8 years in the NutriNet-Santé cohort.

Cumulative hazards were estimated for low (<52), moderate (≥52 and ≤71) and high (>71) impulsivity categories using the Barratt Impulsiveness Scale 11 questionnaire.

Main model was adjusted for baseline age (time-scale), sex, educational level (less than high school degree, <2 years after high school degree, ≥2 years after high school degree), smoking intensity (packs/day), physical activity (International Physical Activity Questionnaire: low, moderate, high), energy intake excluding alcohol (kcal/day), alcohol intake (g/day), diet quality (simplified Programme National Nutrition Santé - Guidelines Score 2), BMI (kg/m^2^), hypertension prevalence or medication (no, yes), hypercholesterolemia prevalence or medication (no, yes), hypertriglyceridemia prevalence or medication (no, yes), and T2D prevalence or medication (no, yes).

**References**

1. Steinberg L, Albert D, Cauffman E, et al. Age differences in sensation seeking and impulsivity as indexed by behavior and self-report: Evidence for a dual systems model. *Dev Psychol* 2008; 44: 1764–1778.

2. Moustafa AA, Tindle R, Frydecka D, et al. Impulsivity and its relationship with anxiety, depression and stress. *Compr Psychiatry* 2017; 74: 173–179.

3. Mozaffarian D, Wilson PWF, Kannel WB. Beyond Established and Novel Risk Factors. *Circulation* 2008; 117: 3031–3038.

4. Cross CP, Copping LT, Campbell A. Sex differences in impulsivity: A meta-analysis. *Psychol Bull* 2011; 137: 97–130.

5. Regitz-Zagrosek V, Gebhard C. Gender medicine: effects of sex and gender on cardiovascular disease manifestation and outcomes. *Nat Rev Cardiol* 2023; 20: 236–247.

6. Schwartz JA, Connolly EJ, Alsolami A. Within-Individual Changes in Impulsivity and Sensation Seeking from Childhood to Early Adulthood and Educational Attainment. *J Youth Adolesc* 2022; 51: 2190–2204.

7. Winkleby MA, Jatulis DE, Frank E, et al. Socioeconomic status and health: how education, income, and occupation contribute to risk factors for cardiovascular disease. *Am J Public Health* 1992; 82: 816–820.

8. Nance R, Delaney J, McEvoy JW, et al. Smoking intensity (pack/day) is a better measure than pack-years or smoking status for modeling cardiovascular disease outcomes. *J Clin Epidemiol* 2017; 81: 111–119.

9. Kale D, Stautz K, Cooper A. Impulsivity related personality traits and cigarette smoking in adults: A meta-analysis using the UPPS-P model of impulsivity and reward sensitivity. *Drug Alcohol Depend* 2018; 185: 149–167.

10. Bos J, Hayden MJ, Lum JAG, et al. UPPS-P impulsive personality traits and adolescent cigarette smoking: A meta-analysis. *Drug Alcohol Depend* 2019; 197: 335–343.

11. Castañer M, Aiello S, Prat Q, et al. Impulsivity and physical activity: A T-Pattern detection of motor behavior profiles. *Physiol Behav* 2020; 219: 112849.

12. Craig CL, Marshall AL, Sjöström M, et al. International physical activity questionnaire: 12-country reliability and validity. *Med Sci Sports Exerc* 2003; 35: 1381–95.

13. Bénard M, Bellisle F, Kesse-Guyot E, et al. Impulsivity is associated with food intake, snacking, and eating disorders in a general population. *Am J Clin Nutr* 2019; 109: 117–126.

14. Franco M, Ordunez P, Caballero B, et al. Impact of Energy Intake, Physical Activity, and Population-wide Weight Loss on Cardiovascular Disease and Diabetes Mortality in Cuba, 1980 2005. *Am J Epidemiol* 2007; 166: 1374–1380.

15. Klatsky AL. Alcohol and cardiovascular diseases: A historical overview. *Ann N Y Acad Sci* 2002; 957: 7–15.

16. Ronksley PE, Brien SE, Turner BJ, et al. Association of alcohol consumption with selected cardiovascular disease outcomes: a systematic review and meta-analysis. *BMJ* 2011; 342: d671–d671.

17. Chaltiel D, Adjibade M, Deschamps V, et al. Programme National Nutrition Santé – guidelines score 2 (PNNS-GS2): development and validation of a diet quality score reflecting the 2017 French dietary guidelines. *British Journal of Nutrition* 2019; 122: 331–342.

18. Gómez-Martínez C, Babio N, Júlvez J, et al. Impulsivity is longitudinally associated with healthy and unhealthy dietary patterns in individuals with overweight or obesity and metabolic syndrome within the framework of the PREDIMED-Plus trial. *International Journal of Behavioral Nutrition and Physical Activity* 2022; 19: 101.

19. Pan A, Lin X, Hemler E, et al. Diet and Cardiovascular Disease: Advances and Challenges in Population-Based Studies. *Cell Metab* 2018; 27: 489–496.

20. Lassale C, Castetbon K, Laporte F, et al. Validation of a Web-based, self-administered, non-consecutive-day dietary record tool against urinary biomarkers. *British Journal of Nutrition* 2015; 113: 953–962.

21. Emery RL, Levine MD. Questionnaire and behavioral task measures of impulsivity are differentially associated with body mass index: A comprehensive meta-analysis. *Psychol Bull* 2017; 143: 868–902.

22. Van Gaal LF, Mertens IL, De Block CE. Mechanisms linking obesity with cardiovascular disease. *Nature* 2006; 444: 875–880.

23. Touvier M, Méjean C, Kesse-Guyot E, et al. Comparison between web-based and paper versions of a self-administered anthropometric questionnaire. *Eur J Epidemiol* 2010; 25: 287–296.

24. Lassale C, Péneau S, Touvier M, et al. Validity of Web-Based Self-Reported Weight and Height: Results of the Nutrinet-Santé Study. *J Med Internet Res* 2013; 15: e152.

25. Fuemmeler BF, Østbye T, Yang C, et al. Association between attention-deficit/hyperactivity disorder symptoms and obesity and hypertension in early adulthood: a population-based study. *International Journal of Obesity 2011 35:6* 2010; 35: 852–862.

26. Sutin AR, Terracciano A, Deiana B, et al. Cholesterol, triglycerides, and the Five-Factor Model of personality. *Biol Psychol* 2010; 84: 186–191.

27. Testa G, Mora-Maltas B, Camacho-Barcia L, et al. Transdiagnostic Perspective of Impulsivity and Compulsivity in Obesity: From Cognitive Profile to Self-Reported Dimensions in Clinical Samples with and without Diabetes. *Nutrients* 2021; 13: 4426.

28. Emery RL, Levine MD, Creswell KG, et al. Impulsivity and midlife cardiometabolic risk: The role of maladaptive health behaviors. *Health Psychology* 2020; 39: 642–654.

29. Lloyd-Jones DM, Nam B-H, D’Agostino, Sr RB, et al. Parental Cardiovascular Disease as a Risk Factor for Cardiovascular Disease in Middle-aged Adults. *JAMA* 2004; 291: 2204.

30. Fields SA, Schueler J, Arthur KM, et al. The Role of Impulsivity in Major Depression: A Systematic Review. *Curr Behav Neurosci Rep* 2021; 8: 38–50.

31. Hare DL, Toukhsati SR, Johansson P, et al. Depression and cardiovascular disease: a clinical review. *Eur Heart J* 2014; 35: 1365–1372.

32. Fuhrer R., Rouillon F. La version française de l’échelle CES-D (Center for Epidemiologic Studies-Depression Scale). Description et traduction de l’échelle d’autoévaluation. *Psychiatry and Psychobiology* 1989; 4: 163–166.
